# The importance of patient-reported outcomes: A call for their integration in the routine care of patients with multiple sclerosis

**DOI:** 10.1177/13524585251349354

**Published:** 2025-06-29

**Authors:** Samantha Cruz Rivera, Stephanie Buxhoeveden, Olalekan Lee Aiyegbusi, Nina Bozinov, Paul Kamudoni, Robert McBurney, Melanie Calvert

**Affiliations:** Centre for Patient Reported Outcomes Research, Institute of Applied Health Research, College of Medical and Dental Sciences, University of Birmingham, Birmingham, UK; Birmingham Health Partners Centre for Regulatory Science and Innovation, University of Birmingham, Birmingham, UK; Virginia Commonwealth University, Richmond, VA, USA; Member Board of Trustees, National Multiple Sclerosis Society, NY, USA; Centre for Patient Reported Outcomes Research, Institute of Applied Health Research, College of Medical and Dental Sciences, University of Birmingham, Birmingham, UK; Birmingham Health Partners Centre for Regulatory Science and Innovation, University of Birmingham, Birmingham, UK; National Institute for Health and Care Research (NIHR) Birmingham Biomedical Research Centre, University of Birmingham, Birmingham, UK; NIHR Applied Research Collaboration (ARC) West Midlands, University of Birmingham, Birmingham, UK; NIHR Blood and Transplant Research Unit (BTRU) in Precision Transplant and Cellular Therapeutics, University of Birmingham, UK; Kootenai Clinic Neurology, Kootenai Health, University of Washington/WWAMI Idaho, Coeur d’Alene, ID, USA; Merck Healthcare KGaA, Darmstadt, Germany; Optimal Healthcare Outcomes, Mashpee, MA, USA; Centre for Patient Reported Outcomes Research, Institute of Applied Health Research, College of Medical and Dental Sciences, University of Birmingham, Birmingham, UK; Birmingham Health Partners Centre for Regulatory Science and Innovation, University of Birmingham, Birmingham, UK; National Institute for Health and Care Research (NIHR) Birmingham Biomedical Research Centre, University of Birmingham, Birmingham, UK; NIHR Applied Research Collaboration (ARC) West Midlands, University of Birmingham, Birmingham, UK; NIHR Blood and Transplant Research Unit (BTRU) in Precision Transplant and Cellular Therapeutics, University of Birmingham, UK

**Keywords:** Patient-reported outcomes, PROs, multiple sclerosis, MS, routine care

## Abstract

Multiple sclerosis (MS) is a chronic inflammatory disease of the central nervous system. Common symptoms include physical disability, cognitive impairment, spasticity, fatigue, bowel/bladder dysfunction and depression. The use of patient-reported outcomes (PROs) can be used to systematically assess the burden of the disease and its impact on treatment and health-related quality of life. Assessment of PROs in routine care of people with MS provides a unique and personal insight into the multifaceted impact of the disease, while avoiding perpetuating the paternalistic medical approach that privileges objective medical measurement.

This review highlights the potential benefits of integrating PROs in MS routine care from the patient and clinician perspective, describes challenges associated with PRO data collection and their use in routine settings. Challenges in the integration of PROs into routine care include (1) selection of PRO measures, (2) provider and technical workflow and (3) equity, diversity and inclusivity challenges. In addition, the review provides solutions and recommendations to facilitate the integration of PROs in MS care. The adoption of PROs in MS routine care is essential to improve MS individuals’ quality of care and deliver patient-centred care, which can lead to increased treatment adherence and patient outcomes.

## Introduction

In 2020, 2.8 million people worldwide were living with multiple sclerosis (MS), a disabling neurological disease with a severe impact on the physical, mental, and social health.^1^ Common symptoms include physical disability, cognitive impairment, spasticity, fatigue, bowel/bladder dysfunction and depression.^2,3^

**Figure 1.**
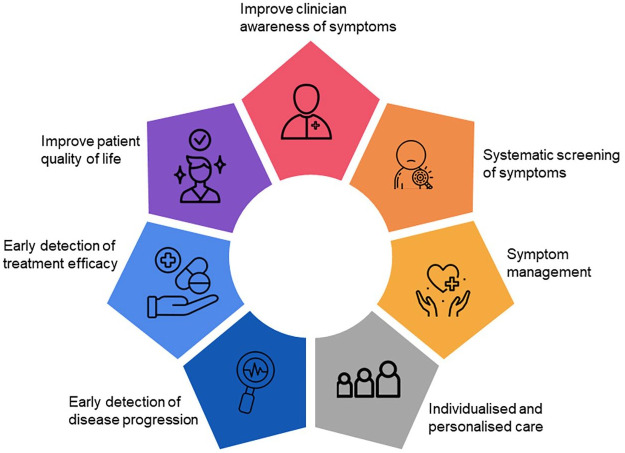
Use of PROs in MS routine care.

The symptomatic burden, impact of disease and treatment, on health-related quality of life (HRQoL) can be systematically assessed via patient-reported outcomes (PROs). PROs are directly reported by patients, using questionnaires without clinician or others’ interpretation.^4^ Assessment of PROs in routine care of people with MS (PwMS) provides a unique and personal insight into the multifaceted impact of MS. PROs have the potential to inform systematic screening and detection of symptoms, tailoring symptom management strategies, inform individualised and personalised care and improve clinician awareness of symptoms that matter to patients.^5,6^ PROs can foster and empower partnership between patients and clinicians, leading to a positive effect on treatment adherence, patient outcomes and enhance patient experience.^7^ Thus, systematic PRO data collection may allow for earlier detection of clinical changes, leading to an opportunity for intervention such as lifestyle, rehabilitation or pharmacological changes. In addition, symptom detection through PROs may allow for timely reduction in unnecessary treatments and lowering of healthcare costs (Figure 1).

In routine care, inclusion of PRO data can enhance clinician assessments by providing information on symptomatic adverse events, physical function and disease-related symptoms, often overlooked in clinical assessment. Furthermore, significant variability of MS symptoms from individual to individual underpins the need for individualised and personalised care, which should be responsive to people’s different and changing course of their diagnosis and ongoing treatment. A personalised approach enables a combination of pharmacological and non-pharmacological treatment that may improve symptoms, stop the progression of the disease and/or reverse the damage of the central nervous system.^8^ Thus, incorporating PROs in routine care should be considered to address patient’s unique needs, avoiding perpetuating the paternalistic medical approach that privileges objective clinical measurement (i.e. clinical observation, diagnostic tests and imaging).^7^ A recent qualitative study showed that incorporating PROs that capture concepts of sexual, bladder and bowel issues into routine practice would be beneficial for discussion during neurologist consultations.^9^ Furthermore, a study comparing the SF-36 domains most valued by PwMs and clinicians found that patients prioritise general health, vitality, mental health and emotional well-being, while clinicians prioritise physical disability, pain and social function.^10^ In the United Kingdom, patients undergoing hip and knee replacement complete PRO questionnaires before and after surgery. The adoption of PROs in clinical practice provide data to compare performance, quality of care and PRO scores between regional providers.^11^

Despite the benefits of PROs, there are different challenges in their integration into routine care including (1) selection of PRO measures, (2) provider and technical workflow and (3) equity, diversity and inclusivity challenges. Additional challenges include administrative and technical workflow issues, existing of too many PRO instruments for almost every symptom, a lack of consensus on which PRO instrument to use, lack of patient engagement, patient’s concerns related to data privacy and burden, which can lead to missing PRO information.^6,12,13^

Thus, the aim of this paper is to highlight the benefits of integrating PROs in MS routine care from the patient and clinician perspective, describe challenges associated with PRO data collection and use in routine settings and provide solutions and recommendations to facilitate the integrations of PRO in MS care.

## Value of PROs to stakeholders

The value of PROs has been increasingly recognised beyond PwMS by healthcare professionals, regulators and policymakers. Box 1 provides patients’ and clinicians’ perspectives of incorporating PROs in MS routine care.

**Box 1.** Stakeholder’s Perspective on the Value of PROs in MS Routine Care.

**Patients’ perspective:** • Empower patients by monitoring their symptoms, disease-related challenges, and progress. • Empower patients to request alternative treatment and communicate their preferences. • Empower patients to ask questions about their health and disease-related challenges. • Increased involvement in care planning and decision-making. • Increase awareness of expected treatment outcomes. • Improve efficiency of clinical appointments. **Clinicians’ perspective:** • Prioritisation of MS symptom management. • Provide individualised treatment approach according to patient’s symptoms and stage of the disease. • Improve clinician-patient communication. • Facilitate evaluation of clinic care by using benchmarking or quality metrics. **Example of how PRO data collection inform MS care, based on clinical expert feedback** Clinical expertise suggests that occasionally patients experience clinical deterioration, even though neurological tests and MRIs indicate that their condition remains stable. However, annual PRO data often show a worsening of scores over time, suggesting progression independent of relapse activity. In another scenario, PRO data obtained at the time of diagnosis may show improvement following the initiation of MS clinical care through intervention, reflecting the positive impact of the intervention. For instance, a patient in their 50s reported worsening of ambulation over a 13 month period, despite stable MRIs results. On review of their PRO data, there was a noticeable decline in physical functioning. Despite being on a disease modifying therapy, concerns about progression independent of relapse activity, prompted the initiation of additional rehabilitation services, and different treatment options were discussed. Similarly, a patient in their 30s reported a significant increase in their fatigue levels, despite optimised lifestyle and symptomatic management. On review of their fatigue scores over 3 years, it was identified a clear shift in fatigue, which coincided with the start of a new medication. As a result, the medical team adjusted their MS treatment, which led to an improvement in fatigue scores.

Benchmarking in clinical practice involves assessing areas of best practice and identifying potential areas of improvement. An example of benchmarking is the National Health Service (NHS) in England with the national PROM programme for hip and knee replacement.^14^ PRO data can facilitate real-world evidence (RWE) generation beyond clinical trials. The Food and Drug Administration (FDA)’s RWE Programme emphasise incorporating PROs in RWE generation as a valuable source to support clinician-focused evidence, enhancing patient centricity in drug development.^15,16^ Although randomised controlled trials, observational studies and real-world data (RWD) are complementary to inform clinical practice, there are specific questions that RWD can only answer. In the context of MS, RWD are used to address behavioural, prognosis, and treatment queries.^17^ For instance, understanding predictors of long-term disability, evaluation of the performance of diagnostic criteria in diverse populations, the long-term effectiveness of MS interventions, comparative safety and tolerability between treatments.^17^ Thus, the use of RWD has the potential to improve the quality of care provided to PwMS. The Multiple Sclerosis Data Alliance (MSDA) is a global, multi-stakeholder collaboration focused on advancing research and innovation care for PwMS by enhancing the utilisation of RWD.^18^

## PRO instruments to assess MS symptoms

Various PROMs are available to assess key concepts like symptomatic adverse events, physical function and disease-related symptoms, which are crucial for understanding an intervention’s impact on HRQoL. For example, the Symptom Burden Questionnaire^19^ and the Patient Reported Outcomes Measurement Information System (PROMIS)^20^ physical function assessment are examples of PRO instruments that can be adapted to different diseases and therapy contexts.^21^ Each instrument comprises an item library and questions, from which a specific subset can be chosen to address concepts that matter to the population.

Disease-specific PRO instruments provide valuable information on symptom range, severity and functional impact.^22^ These measures allow comparison across patients with the same condition; however, they do not allow comparison between diseases. Data obtained by disease-specific PROMs can influence clinical decision-making and patient-centred care.^23^ Besides the use of PROs to assess and monitor MS symptoms such as pain, fatigue and mood changes, clinician-reported tools are used to assess additional symptoms such as bladder and bowel dysfunction and cognitive impairment.

Table 1 presents the different patient tools used to assess some common MS symptoms and treatments available to manage the symptoms. The list of symptoms and tools to assess these were drawn from a recent systematic review conducted by Cruz Rivera et al,^24^ Khurana et al,^25^ and the National MS Society webpage.^26^ The PROMs presented below are specific to the MS symptoms listed and are not exhaustive.

**Table 1.** Assessment Tools and Treatments for Common MS Symptoms.

Symptom	Tools to assess symptoms	Pharmaceutical treatment to manage symptoms	Non-pharmaceutical treatment to manage symptoms
Bladder dysfunction^27^	• Urinary Symptom Profile^28^ • Bladder Control Scale • Neuro-QoL^29^ • Functional Assessment of Multiple Sclerosis (FAMS)^30^	• Oxybutynin (Oxytrol, Ditropan XL, Gelnique) • Trospium (Trosec, MAR-Trospium) • Botox • Tolterodine (Detrol) • Prazosin • Mirabregon	• Avoid irritants such as caffeine and alcohol • Pelvic floor physical therapy
Bowel dysfunction^16,27^	• Neurogenic bowel dysfunction score • Bowel Control Scale • Hamburg Quality of Life Questionnaire in Multiple Sclerosis (HAQUAMS)^31^	• Laxatives and stool softeners • Enemas and suppositories	• Avoid irritants such as caffeine and alcohol • Fluid intake • High fibre diet • Fibre supplements • Liquid sugar concentrates
Cognitive symptoms^a^	• Cognitive Change Scale (CCS)^32^ • Multiple Sclerosis Impact Scale (MSIS-29)^33^ • Fatigue Scale for Motor and Cognition (FSMC) • Multiple Sclerosis Quality of Life-54 (MSQOL-54)^34^	• No pharmacological treatment is suggested	• Exercise • Sleep
Depression and anxiety^27,35^	• Beck Depression Inventory (BDI)^36^ • Hospital Anxiety and Depression Scale (HADS)^37^ • Centre for Epidemiological Studies-Depression Scale (CES-D)^38^ • Neuro-QoL^29^ • Leeds Multiple Sclerosis Quality of Life scale (MSIS-29) • Functional Assessment of Multiple Sclerosis (FAMS)^30^ • Hamburg Quality of Life Questionnaire in Multiple Sclerosis (HAQUAMS)^31^ • Multiple Sclerosis International Quality of Life questionnaire (MusiQoL) • Patient-reported outcome indices for multiple sclerosis (PRIMUS) • Multiple Sclerosis Quality of Life-54 (MSQOL-54)^34^	• Citalopram • Duloxetine hydrochloride • Venlafaxine • Paroxetine • Fluoxetine • Bupropion • Sertraline	• Cognitive behavioural therapy • Counselling. • Mindfulness • Exercise
Fatigue^27,35,39^	• Modified Fatigue Impact Scale (MFIS)^40^ • Fatigue Impact Scale (FIS)^41^ • Fatigue Severity Scale (FSS)^42^ • Fatigue Symptoms and Impacts Questionnaires • Relapsing-Remitting MS (FSIQ-RRMS)^43^ • Fatigue Scale for Motor and Cognitive Functions (FSMC)^44^ • Neuro-QoL^29^ • Leeds Multiple Sclerosis Quality of Life scale (MSIS-29) • Functional Assessment of Multiple Sclerosis (FAMS)^30^ • Hamburg Quality of Life Questionnaire in Multiple Sclerosis (HAQUAMS)^31^	• Amantadine • Modafinil • Fluoxetine • Methylphenidate • Dextroamphetamine and amphetamine	• Aerobic exercise • Fatigue management • Energy conservation • Cognitive behavioural therapy • Mindfulness
Spasticity	• EQ-5D	• Baclofen • Botox • Dantrolene • Clonazepam • Diazepam • Tizanidine	• Stretching • Exercise
Sexual dysfunction^45^	• Multiple Sclerosis Intimacy and Sexuality Questionnaire (MSISQ-19)^46^ • Female Sexual Function Index (FSFI)^47^ • Female Sexual Distress Scale (FSDS)^48^ • Neuro-QoL^29^ • Functional Assessment of Multiple Sclerosis (FAMS)^30^ • Multiple Sclerosis International Quality of Life questionnaire (MusiQoL) • Multiple Sclerosis Quality of Life-54 (MSQOL-54)^34^	• PDE-5 (phosphodiesterase-type5) inhibitors • Prostaglandin	• Vacuum devices • Vibrators • Lubricants • Cognitive behavioural therapy • Couples therapy/counselling

a Cognitive impairment is normally measured through clinician reported outcomes such as Brief Repeatable Battery of Neuropsychological (BRB), Brief International Cognitive Assessment for MS (BICAMS), Paced Auditory Serial Addition Test (PASAT) and Stroop Test.

## Barriers to PRO inclusion in MS routine care

Despite the benefits of PROs, there are several challenges with their integration into the care of PwMS. Challenges in monitoring patients’ symptoms and HRQoL through PROMs range from logistics to methodological.

### Selection of PRO measures

One of the main challenges in the selection of PRO measures in MS is the lack of consensus on what instruments should be used. A systematic review identified 405 PRO instruments available to assess MS, of which 82 (20%) were classified as MS-specific and the rest were non-MS-specific.^25^ The large number of existing PRO instruments challenges the comparison of data to assess the effectiveness or quality of care across different MS clinics.

#### Invisible symptoms and stigma

A further problem related to PRO selection is the absence of PRO measures that capture concepts that are meaningful to PwMS. Invisible or hidden symptoms like fatigue, mood and cognitive changes, sexual dysfunction, anxiety and depression are often not captured. Many PwMS experience invisible symptoms, failing to measure what matters to patients can have cumulative effects on additional MS symptoms and negatively impact people’s quality of life.^49^ A recent systematic review assessing the effect of disease modifying therapies on fatigue in MS determined that only 5% of trials of DMTs assessed fatigue as an outcome.^24^ Invisible symptoms suffered by PwMS are frequently exacerbated by the stigma associated with the condition. Individuals with MS, who experience social stigma present higher rates of depression, lower quality of life and work productivity loss.^50^ In addition, stigma affects individuals’ social interactions, leading to their isolation and sense of belonging.^50,51^ Thus, stigma has a negative impact on patients’ overall HRQoL; however, this burden is not routinely recorded in clinical practice. Routine monitoring and discussion of invisible symptoms and stigma between patients and healthcare professionals is paramount to manage symptom and develop strategies to address and mitigate stigma.^49,50^

#### Psychometric properties

Validity, reliability, and responsiveness in PROMs are essential for measures to be clinically useful. Clinically meaningful results determine whether a treatment is beneficial and for interpreting and adopting these findings in clinical decision-making.^52^ Interpretation of PRO data should extend beyond statistical significance as statistically results are not always clinically relevant. Minimal clinically importance difference (MCID) refers to the smallest change in treatment outcome that would be considered significant by PwMS and clinicians.^53^ MCID data helps in determining the effectiveness of a treatment and its impact on patient’s well-being. However, MCID values are not clearly defined for certain PRO measures in MS, like the SF-12, FSQI-RMS and FIS score.^24,54^ MCID values are typically derived from statistical analyses of population averages or distributions, making them useful as a standard for group-level changes rather than reflecting individual patient characteristics or preferences.^55^ Therefore, MCID values may not be appropriate tools for managing the care of PwMS.^56^

Another challenge is the failure to use translated and culturally validated PROMs, leading to sample attrition and missing data due to cultural misinterpretation, ultimately threatening the validity and Generalisability of research findings for clinical practice.^57^ For example, in some cultures admitting to pain is considered ‘weak’, making the numerical scale an unreliable cross-cultural assessment tool, resulting in inaccurate data and undertreated symptoms.^58^ Existing guidelines provide recommendation to ensure the translation process is rigorous and meet cross-cultural equivalence.^59,60^ A systematic review assessing the impact of DMTs on fatigue in MS trials highlighted the lack of administration of culturally validated items when these were available.^24^ The review also noted that some trials used PROMs in English, thereby excluding non-English speaking participants despite recruiting from countries with diverse languages.

#### Response shift

Research has shown that the magnitude of change of MS-specific PRO instruments is small, which can be attributed to response shift.^61^ Response shift refers to a change in one’s self-assessment as a consequence of a change in (1) the respondent’s internal standards of measurement, (2) respondent’s value or (3) redefinition of the construct.^62^ This has important implications on the interpretation of treatment effects as changes in QoL may be a response shift reflection, a treatment effect, or a combination of both.

Response shift is expected among PwMS as the disease course is characterised by its unpredictable and fluctuating nature, impacting different symptoms. These shifts reflect the evolving nature of their health experience over time. For instance, their meaning of severe fatigue may be affected by an acute relapse. Based on that experience, patient’s meaning of ‘severe’ may change along with their priorities regarding QoL and conceptualisation of HRQoL.^61^ In other words, individuals’ PRO responses may vary not only due to changes in their QoL but also due to their understanding of what QoL means to them. Thus, response shift may interfere with the interpretation of PRO scores and detect changes, making it difficult for clinicians and researchers to determine the effectiveness of an intervention.^63,64^

### Provider and technical workflow challenges

The adoption of PROs in routine care can be hampered by logistic and workflow barriers such as the lack of adequate infrastructure, financial resources to enable PRO collection, insufficient staff to address concerns identified by the PRO instruments and increased burden on staff and patients to complete PRO data.^65–67^ Reasons why PRO data may not be integrated into care can be attributed to clinician’s lack of understanding on how to interpret PRO data, perceived lack of value and/or relevance, and insufficient time to interpret and act upon outcomes during clinic appointments.^66–69^ In addition, PwMS may not be motivated to engage with PROMs if they have taken the time to complete them in the past, but felt the results were not incorporated into their care.^6,67^

The inclusion of PRO data in routine care requires their seamless integration with electronic health records (EHR).^66,70^ However, technology to support the integration requires further development. Currently, different EHR have limited or absent capacity for direct input and they require to have a patient-friendly interface and be available in different languages to avoid the exclusion of patients.^6,66^ Data access and confidentiality are further concerns for patients arising from electronic PRO data collection.^69^ Patients may be unsure who will have access to their data and with what purpose, how their privacy will be protected and how PRO data will be processed and used to inform their care.

### Equality, diversity, and inclusivity challenges

A key barrier to PRO data collection is the exclusion of individuals due to unequal access to technology, low computer literacy or learning disabilities.^71,72^ Patients should be provisioned with the device(s) for PRO data collections and receive appropriate training. In addition, individuals should be given the opportunity to choose from a range of modes of delivery (e.g. paper, smartphone applications, web-based completion, telephone interviews, interactive voice response or audio-computer-assisted interviews), to accommodate different levels of literacy, technological proficiency, and personal and cultural requirements.^12,72^

PwMS often face specific challenges in completing PRO measures due to MS-specific disability such as visual problems, cognitive and manual dexterity impairment (e.g. hand dysfunction). As MS progresses, these symptoms progress, making PRO completion burdensome while hindering PRO data collection.^72,73^ Cognitive impairment can prevent individuals to recall past events or choose accurate responses, as PRO measures often rely on memory and attention.^74^ The lack of cognitive accessibility in the development of PRO measures can result in the exclusion of certain PwMS from the regular monitoring of their health and well-being provided by PROs.^72^ The scores of PRO instruments posing excessive requirements, may reflect the cognitive ability to meet the PROM requirements rather than reflecting the patients’ healthcare experiences and outcomes.^75^ To accommodate individual’s cognitive impairment, research has suggested the use of short questionnaires.^12,76^ However, a review involving PwMS evaluating the relationship between participant burden and questionnaire length, recommended selecting PROMs based on outcome content rather than only on the length of the instrument.^77^ The use of proxy-reported measures should be considered as a solution among individuals with advanced cognitive decline.^72^

Expanding PRO completion among PwMS can promote inclusivity and enhance the accuracy and applicability of results.^72,73^ To achieve this, patients’ health status and disability should guide PRO selection and administration,^12^ as the mode of PRO administration and setting of data collection influence self-reporting. Research suggests that administering PROMs at home before clinical appointments is more effective, as it avoids the pressure of busy clinics and potential disturbance from medical personnel.^12^ Completion of PROMs at home is more likely among PwMS with advanced disabilities. Furthermore, individuals may need additional assistance from a caregiver to see, read, or understand the PROMs, depending on the degree of their visual or cognitive impairment.

A further barrier to equal access to treatment is socioeconomic status. A recent study demonstrated that individuals who are socioeconomic deprived with relapsing-remitting MS present higher mortality rates from symptom onset.^78^ Thus, unequal access to treatment may contribute to higher mortality rates, as timely treatment has the potential to delay disability onset.^79^ In addition, a recent systematic review of MS clinical trials of DMTs showed that only 10%–20% of trial participants were recruited from minority groups.^24^ Cohort studies in the United States and United Kingdom have identified higher MS prevalence in certain racial and ethnic groups, suggesting that treatment effectiveness tested primarily in White participants may not be generalisable to the entire MS population.^80,81^

A further barrier is the lack of knowledge and understanding of PRO data completion. Research have shown that patients often feel some PROM questions are irrelevant, participants were not informed about what PROs are or the value of PRO completion.^68^ Therefore, it is important to provide training and education to PwMS to ensure they understand the value and use of PRO data in their care.

## Key considerations to successfully incorporate PROs in MS routine care

Accelerating the implementation of PROs in routine care requires addressing administrative, system, clinician, and patient-level. Facilitators at the clinician-level include providing training on the importance of PRO data to healthcare providers and training to familiarise clinical staff with PRO system features.^82–84^ Additional facilitators include co-designing PRO systems with clinicians and patients input to promote integration in the routine workflow, consulting with the clinical team how to best incorporate PROs in the new workflows.^85^ Facilitators at the patient-level include effectively communicating to patients the reason why PRO data is being administered and how the data will be used.^86–88^ To effectively capture outcomes that matter to patients such as ‘hidden symptoms’ and stigma, it is essential to involve patients in selecting PRO measures and consider the characteristics of PRO candidate measure.^12,89,90,91^

Furthermore, considering and addressing respondent burden is crucial to prevent avoidable missing and poor reporting of PRO data. Considerations include offering alternative modes to PROM administration like mail or telephone, interviewer, and interactive voice response completion.^12,72^ Implementing computer adaptative testing (CAT) for electronic PROM collection allows tailoring questionnaires to individual respondents by eliminating irrelevant questions.^83,92,93^ CAT should be considered as a solution to address challenges related to fatigue and concentration in PwMS. A simulation study using multidimensional CAT on the Multiple Sclerosis Quality of Life-54 instrument (MSQoL-54) demonstrated that the instrument retained its precision after removing irrelevant HRQoL items.^94^ To reduce the burden of electronic PROM completion among individuals with lack of digital skills/low computer literacy or advanced disabilities consider staff administered questionnaires as one strategy for broadening the inclusion of patients. The PRO responses should be decided by the respondent, not by the assisting individual.^12^ Finally, when selecting assessment timepoints, it is essential to consider the nature of the condition, potential treatment effects, while considering patients’ and clinicians’ input.^12^

Existing guidelines such as the PROTEUS (Patient-Reported Outcomes Tools: Engaging Users and Stakeholders)-Practice Guide provides guidance on how to design, implement and manage PRO systems and related data in PRO clinical care.^82^ PROTEUS leads the Learning Health Network and PROMs Equity Project, focused on addressing barriers to PROM implementation in clinical practice. Additional guidance includes the PRO Ethics guidelines, highlighting ethical issues to be addressed in PRO clinical research to reduce participant risk and burden. Ethical issues addressed by the guidelines include items related to PRO background and rationale, PRO-specific eligibility requirements, PRO concepts and domains, participant acceptability and burden, administration of PRO questionnaires for participants who are unable to self-report PRO data, input on PRO strategy by patient partners or members of the public, PRO data collection, management and analysis and PRO dissemination plans. The PRO Ethics guidelines are for the use of research groups and ethics committees, including patients, research participants and members of the public.^89^ Additional resources include key considerations to reduce or address respondent burden in PRO data collection and strategies to include underserved groups in PRO clinical trials and routine care.^12,72^ A summary of recommendations for the inclusion of PROs in routine MS are provided in Box 2.

**Box 2.** Summary of Recommendations to Advance PROs in MS Care.

1. Clearly state the objective for PRO data collection and how PRO data will be used to support the care of PwMS 2. Clearly state how PRO data will be used in the care of PwMS and who will have access to the data. 3. Carefully select PROMs, considering measurement properties, patient acceptability among PwMS, and symptom burden. 4. Ensure that PROMs capture concepts that are meaningful to PwMS, including ‘hidden symptoms and stigma. 5. Ensure PROMs and data collection methods are accessible and reflect the diversity of the society. 6. Consider language and cultural validity of PROMs when collecting PRO data from diverse populations. 7. Provide training and support to patients and healthcare providers. 8. Use international standards for including PROs in routine practice (for instance, PROTEUS-Practice Guide and PRO Ethics guidelines).

PROs play a critical role in assessing symptom burden; however, they are often underutilised in MS routine care. Incorporating PROs in MS routine care has the potential to improve quality of care, promote patient-centred care, and improve treatment adherence and patient outcomes. However, it is crucial to ensure that the PRO instruments used are scientifically validated and reliable. In addition, PRO measures and data collection must reflect the needs of PwMS, to ensure equitable and accessible care.
